# Mentalization and dysfunctional eating behaviors: the mediating role of body uneasiness

**DOI:** 10.3389/fpsyg.2026.1812742

**Published:** 2026-05-25

**Authors:** Gelsomina Cecere, Ilaria La Penna, Gianluca Poselek, Emanuele Rocchetti, Michela Ciampricotti, Luigia Simona Sica, Alessandro Frolli

**Affiliations:** 1 Developmental Neuroscience Research Lab (DNR Lab), Department of International Humanities and Social Sciences, Rome University of International Studies, Rome, Italy; 2 Department of Humanities, University of Naples Federico II, Naples, Italy

**Keywords:** body image distress, body uneasiness, eating behaviors, eating disorders, mediation model, mentalization, reflective functioning

## Abstract

**Introduction:**

Adolescence is a vulnerable period with heightened risk of dysfunctional eating attitudes and behaviors; the mechanisms linking mentalization difficulties to eating behavior via body uneasiness remain unclear.

**Methods:**

91 Italian adolescents completed the Body Uneasiness Test (BUT), Eating Attitudes Test (EAT), and Mentalization Questionnaire (MZQ). Spearman correlations and bootstrap mediation analysis were conducted using JASP.

**Results:**

Lower mentalization was associated with higher body uneasiness and dysfunctional eating attitudes. Mediation analysis showed that body uneasiness statistically accounted for the association between mentalization difficulties and dysfunctional eating behaviors (indirect effect significant; direct effect non-significant).

**Discussion:**

Body uneasiness may serve as an intermediary process linking self-reported mentalization difficulties to dysfunctional eating patterns in adolescence. Findings are cross-sectional and causal interpretation is not warranted.

## 1 Introduction

Adolescence represents a critical and particularly vulnerable developmental phase, characterized by profound biopsychosocial transformations that simultaneously affect multiple domains of functioning. At the biological level, pubertal changes alter body shape, weight, and physical appearance, directing adolescents’ attention increasingly toward their own bodies (Blos, 1979). At the psychological level, this period involves a progressive redefinition of identity, self-concept, and self-worth, processes that are deeply intertwined with bodily experience (Erikson, 1970). At the social level, adolescents are increasingly exposed to peer evaluation, social comparison, and culturally shared esthetic ideals, which further amplify the salience of physical appearance. Within this context, the body becomes a central object of attention and evaluation. Body image defined as a multidimensional construct encompassing perceptual, cognitive, emotional, and behavioral components related to one’s physical appearance (Cash, 2002) undergoes significant development during adolescence and is particularly susceptible to distortion and dissatisfaction during this stage. The widespread diffusion of idealized and often unattainable esthetic standards, promoted through both traditional mass media and, more recently, social media platforms, intensifies processes of social comparison and internalization of beauty norms (Thompson et al., 1999; Eckler et al., 2017; Aparicio-Martinez et al., 2019; Franchina and Coco, 2018). Such processes contribute to body dissatisfaction, which has been consistently identified as one of the most robust and transdiagnostic risk factors for the development of dysfunctional eating attitudes and behaviors in adolescent populations (Carta et al., 2008; Cafagna et al., 2006).

### 1.1 Dysfunctional eating behaviors and eating disorders

Dysfunctional eating behaviors (DEB) encompass a broad spectrum of maladaptive attitudes and conduct related to food and the body, including dietary restriction, binge eating, excessive preoccupation with weight and body shape, and compensatory behaviors, that do not necessarily meet the full diagnostic criteria for a clinical eating disorder (ED). According to the Diagnostic and Statistical Manual of Mental Disorders (DSM-5-TR; American Psychiatric Association, 2022), EDs such as anorexia nervosa, bulimia nervosa, and binge eating disorder require more stringent criteria of severity, persistence, and functional impairment. Epidemiological data indicate that lifetime prevalence of DSM-5 eating disorders ranges from 5.5 to 17.9% in young women and from 0.6 to 2.4% in young men in Western settings, with particularly elevated rates observed among gender and sexual minority individuals (Silén and Keski-Rahkonen, 2022). Subthreshold DEB are considerably more prevalent in non-clinical adolescent populations and are recognized as significant risk factors for the subsequent development of full-threshold eating disorders (Silén and Keski-Rahkonen, 2022). The present study focuses on DEB in a non-clinical adolescent sample, operationalized through the Eating Attitudes Test (EAT) as a validated screening instrument.

Individuals engaging in DEB frequently experience difficulties in identifying, tolerating, and expressing emotional states, as well as in managing interpersonal relationships (Godart et al., 2004; Weinbach et al., 2018). In this regard, the relational and family context plays a meaningful role: weight-related derogatory comments from family members are associated with greater body dissatisfaction, whereas secure and attuned parental relationships may reduce the onset and maintenance of eating-related difficulties by supporting emotional regulation and distress tolerance (Rothschild-Yakar et al., 2013). This is particularly relevant in light of the theoretical framework adopted in the present study, given that mentalization, understood as the capacity to understand one’s own and others’ mental states, develops primarily within early attachment relationships (Fonagy and Luyten, 2009). Invalidating or poorly attuned relational environments may compromise the development of reflective functioning, rendering adolescents more vulnerable both to body-related distress and to dysfunctional eating patterns. The family context thus constitutes a theoretically coherent background variable within the self-reported mentalization difficulties, body uneasiness, and DEB framework that the present study aims to examine.

### 1.2 Mentalization and its role in eating disorders

In recent years, scientific literature has progressively focused on the sociocognitive processes implicated in the development and persistence of dysfunctional eating behaviors (Oldershaw et al., 2011; Skårderud, 2007; Tchanturia et al., 2004; Tonelli and de Siqueira Rotenberg, 2021). Within this framework, particular emphasis is given to the construct of mentalization, understood as a complex metacognitive capacity that enables individuals to infer and represent their own and others’ intentional mental states, including emotions, thoughts, beliefs, desires, and feelings that guide and explain human behavior (Bateman and Fonagy, 2016). It is a multidimensional construct articulated along different axes: it can manifest in automatic or controlled forms, be oriented toward the self or the other, focus on internal or external aspects, and have a cognitive or affective valence (Bateman and Fonagy, 2016). For mentalization to be effective, it is necessary to maintain a balance among these dimensions and to adapt its modality to the specific context (Fonagy and Luyten, 2009; Luyten et al., 2020).

Individuals engaging in dysfunctional eating behaviors often present difficulties in reading and interpreting their own and others’ mental and emotional states (Jewell et al., 2016; Oldershaw et al., 2011; Skårderud, 2007; Tonelli and de Siqueira Rotenberg, 2021). Their modes of mentalization tend to remain primitive, characterized by the inability to distinguish between physical and emotional states, as well as between self and others’ experiences, and by a failure to attribute causality to both personal and interpersonal experiences. These difficulties are associated with the use of dysfunctional eating behaviors as a mode of expressing and manifesting emotions and thoughts that cannot otherwise be represented (Skårderud, 2007). Furthermore, individuals with eating disorders often show greater uncertainty regarding their own mental states and more marked alexithymia, although their capacity to read others’ mental states may be partially preserved (Bachar et al., 2010; Kjaersdam Telléus et al., 2024). In such cases, mental states that cannot be represented as ideas or feelings are instead transferred to the bodily level through food control or concrete actions, a phenomenon described as embodied mentalization, in which the body becomes the container of experiences that cannot be processed through other channels (Luyten et al., 2012; Skårderud, 2007). Weinbach et al. (2018) highlighted a significant association between eating disorders and difficulties in emotional regulation, noting that individuals across different eating disorder presentations frequently experience challenges in developing emotional and affective awareness. The transdiagnostic model proposed by Fairburn et al. (2003) similarly identifies the mechanisms that maintain eating disorders, including clinical perfectionism, low self-esteem, interpersonal difficulties, and emotion intolerance, suggesting that individuals with low emotional tolerance may regulate their internal states by resorting to dysfunctional eating behaviors as a means to reduce emotional suffering and restore psychological balance.

### 1.3 Body uneasiness as a mediating construct

A key variable in understanding the association between self-reported mentalization difficulties and dysfunctional eating behaviors is body uneasiness. Body uneasiness is conceptualized as a multidimensional experience of distress related to body image, encompassing cognitive, emotional, and behavioral components, including weight phobia, body image concerns, avoidance, compulsive self-monitoring, and depersonalization (Cuzzolaro et al., 2006). Although body dissatisfaction has long been recognized as a transdiagnostic predictor of eating-related psychopathology (Carta et al., 2008), the specific construct of body uneasiness has received comparatively less attention in studies examining its role alongside self-reported mentalization difficulties. Adolescents with greater self-reported difficulties in reflective functioning may be less able to symbolize internal states through language or more elaborated mental processes; in such cases, these states may instead be transferred to the body through embodied mentalization, potentially amplifying distress related to physical appearance and body perception (Luyten et al., 2012; Skårderud, 2007). Consistent with this theoretical account, body uneasiness has been proposed as a plausible intermediary variable through which self-reported mentalization difficulties may be associated with dysfunctional eating patterns, although this hypothesis requires empirical examination in cross-sectional and, ultimately, longitudinal designs.

## 2 The current study

Despite growing evidence linking mentalization difficulties to eating-related psychopathology, the mechanisms through which impaired reflective functioning translates into dysfunctional eating attitudes and behaviors remain insufficiently clarified. In particular, the potential role of body uneasiness as an intermediary process in this association has received limited empirical attention, especially in non-clinical adolescent samples. To address this gap, the present study examined the associations between self-reported mentalization difficulties, body uneasiness, and dysfunctional eating attitudes and behaviors in a sample of Italian adolescents, testing a simple mediation model in which body uneasiness was hypothesized to account for the relationship between mentalization and dysfunctional eating. Body uneasiness was conceptualized as a multidimensional experience of distress related to body image, encompassing cognitive, emotional, and behavioral components including weight phobia, body image concerns, avoidance, compulsive self-monitoring, and depersonalization (Cuzzolaro et al., 2006). This construct was considered especially relevant in adolescence, given the centrality of body-related experiences during this developmental stage. Theoretically, adolescents who struggle to recognize and symbolize internal states through mental representations may instead transfer such states to the body through embodied mentalization (Skårderud, 2007; Luyten et al., 2012), amplifying body-related distress and, in turn, increasing vulnerability to maladaptive eating patterns. Based on this theoretical framework, the following hypotheses were formulated:

*H1*: Lower levels of self-reported mentalization (MZQ) will be associated with higher levels of body uneasiness (GSI): adolescents reporting greater difficulties in reflective functioning are expected to show higher levels of body-related distress.

*H2*: Higher levels of body uneasiness (GSI) will be associated with higher levels of dysfunctional eating attitudes and behaviors (EAT): greater body-related distress is expected to be associated with more marked dysfunctional eating patterns.

*H3*: Body uneasiness (GSI) will statistically mediate the association between self-reported mentalization difficulties (MZQ) and dysfunctional eating attitudes and behaviors (EAT): the association between mentalization difficulties and DEB is expected to be accounted for, at least in part, by body uneasiness as an intermediary variable. In line with the cross-sectional nature of the data, all hypotheses were formulated in terms of statistical associations rather than causal effects.

## 3 Materials and methods

The sample consisted of 103 adolescents recruited from multiple upper secondary schools. Data collection took place in spring 2025 within the framework of a university-led laboratory initiative, through which research staff conducted on-site visits to several schools in the Rome metropolitan area. Participants were asked to complete a digital self-report questionnaire using their personal smartphones.

To ensure sample homogeneity, participants were required to declare: (a) that they did not have neurodevelopmental disorders, (b) that they did not have neurological disorders, and (c) that they did not have psychiatric disorders. For this reason, the questionnaire included closed-ended questions such as: “Do you have any psychiatric disorders?”, “Do you have any neurological disorders?”, and “Do you have any neurodevelopmental disorders?” For each question, in addition to answering “yes” or “no,” participants were asked to specify any conditions, if present.

Initially, 103 participants were recruited; however, 12 were excluded due to reporting the presence of neurological, psychiatric, or neurodevelopmental disorders. The final sample was sex-balanced and therefore consisted of 91 participants, including 45 females and 46 males, aged between 15 and 20 years (mean age = 17.19; SD = 0.84). Sex was assessed using binary biological categories (female/male). Specifically, the sample included 1.09% of students enrolled in the second year of upper secondary school, 26.37% in the third year, 71.42% in the fourth year, and 1.09% in the fifth year.

The study was conducted after obtaining informed consent and in accordance with the ethical standards of the Declaration of Helsinki, as well as with the approval (ID 25/2024) of the Ethics Committee (CERUS) of the Department of International Human and Social Sciences at the International University of Rome (UNINT).

The research protocol was digitized using the Google Forms platform to ensure efficient and user-friendly administration of the questionnaires. The form was completely anonymous to protect participants’ privacy. The protocol consisted of a sequence of three questionnaires, administered in the following order: the Body Uneasiness Test (BUT), the Eating Attitudes Test (EAT), and the Mentalization Questionnaire (MZQ). The entire protocol required approximately 30 min to complete. The data collected through these questionnaires were subsequently analyzed by the Developmental Neuroscience Research Lab, ensuring a rigorous approach to data management and analysis.

### 3.1 Measures

The Body Uneasiness Test (BUT; Cuzzolaro et al., 2006) is a self-report questionnaire developed to assess discomfort associated with body uneasiness, integrating the cognitive, emotional, and behavioral dimensions of such uneasiness. The test consists of 71 items divided into two main sections: BUT-A, which measures five dimensions of body uneasiness—Weight Phobia (WP), Body Image Concerns (BIC), Avoidance (A), Compulsive Self-Monitoring (CSM), and Depersonalization (D); and BUT-B, which investigates specific concerns related to body parts or bodily functions. Responses to the BUT items are given on a six-point Likert scale, where 0 indicates the absence of problems and 5 indicates maximum severity. Higher scores correspond to greater overall body uneasiness. In our study, we chose to administer only the BUT-A section, which provides a Global Severity Index (GSI) and five subscales, described as follows: Weight Phobia (WP) assesses the fear of being or becoming fat, including anxiety related to weight gain and body size (e.g., items #9, #10, #18, #21, #24, #31, #32, #33); Body Image Concerns (BIC) measures anxiety related to physical appearance, encompassing a wide range of concerns beyond weight (e.g., items #3, #4, #6, #12, #15, #22, #23, #25, #34); Avoidance (A) analyses avoidant behaviors related to body uneasiness, revealing coping strategies used to avoid distressing situations (e.g., items #5, #8, #13, #16, #19, #30); Compulsive Self-Monitoring (CSM) explores the obsessive tendency to monitor one’s physical appearance, focusing particularly on body shape, weight, and size (e.g., items #1, #11, #17, #20, #27); Depersonalization (D) measures the sense of detachment and estrangement from one’s body, highlighting disturbances in bodily perception and personal identity (e.g., items #2, #7, #14, #26, #28, #29). The internal reliability of the subscales was assessed using Cronbach’s alpha, with the following values: Weight Phobia: 0.84; Body Image Concerns: 0.90; Avoidance: 0.79; Compulsive Self-Monitoring: 0.82; Depersonalization: 0.85. These values indicate good internal consistency for each test dimension (Cuzzolaro et al., 2006), confirming the reliability of the instrument in assessing body uneasiness. The internal reliability of the subscales was assessed using Cronbach’s alpha, with the following values: Weight Phobia: 0.84; Body Image Concerns: 0.90; Avoidance: 0.79; Compulsive Self-Monitoring: 0.82; Depersonalization: 0.85. These values indicate good internal consistency for each test dimension (Cuzzolaro et al., 2006), confirming the reliability of the instrument in assessing body uneasiness. Within our sample, internal reliability was also evaluated using Cronbach’s alpha, yielding the following values: Total Score: 0.96; Weight Phobia: 0.91; Body Image Concerns: 0.93; Avoidance: 0.86; Compulsive Self-Monitoring: 0.79; Depersonalization: 0.85.

The Eating Attitudes Test (EAT; Garner et al., 1982) is a self-report screening instrument designed to assess dysfunctional eating attitudes and behaviors and to identify individuals with dysfunctional eating attitudes and behaviors. It does not provide a clinical diagnosis. The test consists of 26 items that explore various aspects of eating behavior, including concerns about body weight, calorie restriction, food control, and compensatory behaviors (e.g., self-induced vomiting). Responses to the EAT items are given on a six-point Likert scale, where 1 indicates “never” and 6 indicates “always.” The test includes a total scale and three subscales. Specifically, the first dimension, Dieting, measures the degree of food restriction and the avoidance of foods perceived as fattening (e.g., items #1, #6, #7, #10, #11, #12, #14, #16, #17, #22, #23, #24, #26). The second dimension, Bulimia and Food Preoccupation, assesses the presence of bulimic behaviors and excessive preoccupation with food (e.g., items #3, #4, #9, #18, #21, #25). Lastly, the third dimension, Oral Control, refers to concerns about body weight and physical appearance (e.g., items #2, #5, #8, #13, #15, #19, #20). The total scale demonstrated good reliability (*α* = 0.86). At the subscale level, Dieting showed good internal consistency (*α* = 0.87), Bulimia and Food Preoccupation demonstrated acceptable reliability (*α* = 0.70), whereas Oral Control showed moderate internal consistency (*α* = 0.62). This study used the Italian version of the EAT (Dotti and Lazzari, 1998). The scoring method proposed by the authors also includes a total score, where higher values (cut-off = 20) indicate greater tendencies toward eating disorder symptoms. Within our sample, the total scale also demonstrated good reliability (*α* = 0.85).

The Mentalization Questionnaire (MZQ; Hausberg et al., 2012) is a 15-item tool designed to assess self-reported mentalization difficulties, organized into four distinct dimensions. The first dimension, REF, comprises four items (i.e., #14, #9, #13, #5), which focus on the tendency to interpret others’ mental states from a self-referential perspective (e.g., “If someone yawns in my presence, that’s a reliable sign that he is bored in my company.”) and a tendency to avoid acknowledging one’s own emotions due to the fear of becoming overwhelmed by them. The second dimension, EA, also includes four items (i.e., #10, #11, #15, #8), which emphasize difficulties in recognizing one’s own emotional states (e.g., “Often I don’t even know what is happening inside of me.”). The third dimension, PE, consists of four items (i.e., #12, #1, #4, #7) that highlight an inability to distinguish between a mental state (e.g., a thought) and a real-life event (e.g., “Often I feel threatened by the idea that someone could criticize or offend me.”). Finally, the fourth dimension, RA, includes three items (i.e., #6, #2, #3), which capture difficulties in emotion regulation (e.g., “Often I can’t control my feelings.”). Respondents rate each item on a 5-point Likert scale (from 1 = “I agree” to 5 = “I disagree”), with lower scores reflecting greater self-reported mentalization difficulties. The scoring system proposed by the authors also allows for a total score, where lower values (cut-off = 3.30) reflect greater difficulties in mentalization. The Italian version of the MZQ was used for this study (Raimondi et al., 2022). Cronbach’s alphas: REF = 0.49; EA = 0.71; PE = 0.61; RA = 0.55 (Raimondi et al., 2022). The adolescent version of the Questionnaire (Ponti et al., 2019) was administered for this study. Within our sample, Cronbach’s alpha was assessed, yielding the following values: MZQ_TOT = 0.85; REF = 0.56; EA = 0.70; PE = 0.59; RA = 0.69.

### 3.2 Statistical analysis

All statistical analyses were conducted using the open-access software JASP, version 0.19.3. Descriptive statistics and Spearman correlation coefficients were calculated to examine bivariate relationships among the study variables, including mentalization, body image distress, and dysfunctional eating attitudes. In the present study, the construct “risk of developing dysfunctional eating attitudes” was operationalized through the total score of the Eating Attitudes Test (EAT), treated as a continuous variable. It is important to note that the EAT is a screening instrument and does not provide a clinical diagnosis; therefore, higher scores were interpreted as reflecting greater severity of dysfunctional eating attitudes and an increased levels of dysfunctional eating attitudes and behaviors, rather than the presence of a diagnosed disorder.

For the statistical analyses, the total scores of the MZQ, the GSI, and the EAT were used. The variables did not show a normal distribution. For the variables considered, the distribution indices showed the following values of skewness and kurtosis: for the GSI, skewness was 0.64 and kurtosis was −0.43; for the EAT, skewness was 0.90 and kurtosis was −0.05; finally, for the MZQ, skewness was 0.86 and kurtosis was 1.02.

Before testing the mediation model, preliminary analyses were conducted to verify the necessary conditions for mediation. In line with established recommendations, we examined whether there was a significant association between the independent variable (MZQ_TOT) and the dependent variable (EAT), between the independent variable and the proposed mediator (GSI), and between the mediator and the dependent variable. The results of the correlation analyses showed that all these associations were statistically significant (*p* < 0.05), thus supporting the suitability of the data for mediation analysis.

Finally, a mediation model was tested to examine the role of the severity of symptoms associated with body image distress (GSI) in the relationship between self-reported mentalization difficulties (MZQ_TOT) and the levels of dysfunctional eating attitudes (EAT). Specifically, the severity of symptoms related to body image distress was included as a mediator of the association between MZQ_TOT and EAT. The mediation analysis was conducted using the bootstrap method with 1,000 resamples and bias-corrected percentile confidence intervals. This non-parametric resampling technique provides robust estimates of indirect effects, particularly when sampling distributions may deviate from normality. Standardized path coefficients, standard errors, *z*-values, and *p*-values are reported for all direct, indirect, and total effects.

## 4 Results

### 4.1 Correlation analysis

A series of Spearman correlation analyses were conducted to examine the relationships between self-reported mentalization difficulties (MZQ), body image distress (GSI), and the levels of dysfunctional eating attitudes and behaviors (EAT). The MZQ score, which reflects self-reported difficulties in interpreting one’s own and other’s mental and emotional states, was found to be significantly and negatively associated with the severity of body image distress symptoms (GSI), *r* (91) = −0.640, *p* < 0.001, and with the levels of dysfunctional eating attitudes and behaviors (EAT), *r* (91) = −0.496, *p* < 0.001. Furthermore, GSI and EAT showed a significant positive relationship, *r* (91) = 0.765, *p* < 0.001 (Table 1).

**Table 1:** Correlation between GSI, EAT, MZQ.

Variables	GSI	EAT	MZQ
(1) GSI			
Spearman’s rho	—		
*p*-value	—		
(2) EAT			
Spearman’s rho	0.765***	—	
*p*-value	<0.001	—	
(3) MZQ			
Spearman’s rho	−0.640***	−0.496***	—
*p*-value	<0.001	<0.001	—

^*^p < 0.05; ^**^p < 0.01; ^***^p < 0.001.

### 4.2 Mediation model

Overall, the correlation analysis revealed significant associations between mentalization, body image distress, and the dysfunctional eating attitudes and behaviors. Specifically, mentalization (MZQ) was significantly associated with both body image distress (GSI) and dysfunctional eating attitudes (EAT), and body image distress was significantly associated with dysfunctional eating attitudes (see Table 1). Before testing the mediation model, these preliminary associations were examined to verify that the necessary conditions for mediation were met. The observed pattern of correlations provided empirical support for proceeding with mediation analysis, as the independent variable (MZQ) was significantly related to both the mediator (GSI) and the dependent variable (EAT), and the mediator was significantly associated with the dependent variable. To further explore these relationships, a simple mediation model was tested, using GSI as a mediator of the relationship between MZQ and EAT.

**Figure 1:**
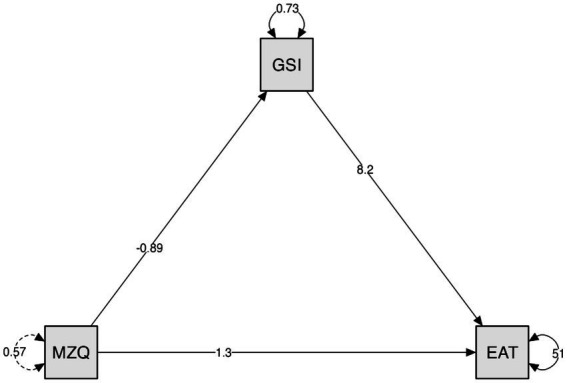
Mediation model depicting the indirect effect of mentalization (MZQ) on eating disorder indicator (EAT) via difficulties in body image distress (GSI).

The total effect of MZQ on EAT was statistically significant, *β* = −6.025, SE = 1.384, *z* = −4.354, *p* < 0.001, 95% CI [−9.269, −3.052]. However, the direct effect of MZQ on EAT, controlling for the mediator, was not significant (*β* = 1.271, *p* = 0.362), suggesting indirect-only mediation; however, given the opposing signs of the direct and total effects, these results should be interpreted with caution. The total indirect effect was significant, *β* = −7.296, SE = 1.245, *z* = −5.861, *p* < 0.001, 95% CI [−9.941, −5.254], indicating that the association between MZQ and EAT is accounted for by the mediator GSI (Figure 1).

## 5 Discussion

The present study examined the associations among self-reported mentalization difficulties, body uneasiness, and dysfunctional eating attitudes in a non-clinical sample of Italian adolescents, testing a mediation model in which body uneasiness accounted for the relationship between mentalization and eating-related psychopathology. Three main findings emerged. First, lower self-reported mentalization was significantly associated with higher levels of body uneasiness. Second, greater body uneasiness was significantly associated with higher scores on the dysfunctional eating attitudes measure. Third, body uneasiness statistically accounted for the association between self-reported mentalization difficulties and dysfunctional eating attitudes. However, given the opposing signs of the total and direct effects, this pattern is more consistent with inconsistent mediation rather than full mediation, and should be interpreted with caution. These findings are discussed in relation to existing theoretical frameworks and their clinical implications.

The significant negative association between self-reported mentalization (i.e., reflective functioning difficulties as measured by self-report) and body uneasiness is consistent with theoretical accounts proposing that difficulties in representing and regulating internal mental states may increase vulnerability to distorted body perception during adolescence. In the present cross-sectional data, adolescents reporting greater self-reported mentalization difficulties showed higher levels of body uneasiness, a pattern consistent with theoretical accounts suggesting that difficulties in representing internal mental states may be associated with increased vulnerability to body-related distress (Skårderud, 2007; Luyten et al., 2012). This interpretation aligns with the concept of embodied mentalization, whereby emotions and thoughts that cannot be symbolized through mental representations are instead transferred to the body, manifesting as body uneasiness or somatic preoccupation (Skårderud and Fonagy, 2011). Adolescence represents a particularly vulnerable developmental window for these dynamics, given the centrality of physical changes, identity redefinition, and exposure to sociocultural pressures regarding body ideals (Blos, 1979; Erikson, 1970).

The significant positive association between body uneasiness and dysfunctional eating attitudes replicates and extends prior findings highlighting the central role of body image distress in eating-related psychopathology (Carta et al., 2008; Oldershaw et al., 2011). Adolescents reporting greater body uneasiness, encompassing weight phobia, body image concerns, avoidance, compulsive self-monitoring, and depersonalization, also reported higher levels of dysfunctional eating attitudes. This result is consistent with the transdiagnostic model proposed by Fairburn et al. (2003), according to which body dissatisfaction and weight-related concerns constitute core maintenance mechanisms across eating disorder presentations. It is worth noting, however, that the high correlation observed between GSI and EAT scores (rho = 0.765) may also reflect a degree of conceptual overlap between the two constructs, as both instruments include items related to weight concerns and eating-related cognitions. A critical issue concerns the substantial conceptual and statistical overlap between body uneasiness (GSI) and dysfunctional eating attitudes (EAT), as reflected by the high correlation observed between these variables (*ρ* = 0.765). Indeed, both instruments include content related to weight concerns, body dissatisfaction, and cognitive preoccupation with physical appearance, which may partially account for their strong association. This raises the possibility that the mediation model may capture the relationship between closely related facets of the same underlying domain of body-related distress, rather than a fully distinct mediational process. However, it is important to note that the two constructs, while related, are theoretically distinguishable. The GSI primarily assesses a broader, multidimensional experience of body uneasiness, encompassing emotional, cognitive, and behavioral aspects of body image distress, whereas the EAT focuses more specifically on dysfunctional eating attitudes and behaviors, including dietary restraint, bulimic tendencies, and control over food intake. From this perspective, body uneasiness may represent a more general vulnerability factor that is associated with, but not identical to, maladaptive eating patterns. Nevertheless, given the degree of overlap between the measures, the present findings should be interpreted with caution. Future research should aim to disentangle these constructs more clearly, for instance by employing multimethod assessments or more differentiated measures, in order to better clarify the specificity of the mediational pathway observed. This limitation is further discussed in the limitations section.

The mediation analysis indicated that body uneasiness accounted for the association between self-reported mentalization difficulties and dysfunctional eating attitudes, with the direct effect of mentalization on eating attitudes becoming non-significant after inclusion of the mediator. These results suggest that the association between self-reported mentalization difficulties and dysfunctional eating attitudes was statistically accounted for by body uneasiness in the model. Nevertheless, the pattern of opposing signs across direct and total effects indicates a complex association that warrants cautious interpretation and replication in longitudinal designs. In the present sample, adolescents reporting greater self-reported mentalization difficulties also showed higher levels of body uneasiness, which was in turn associated with higher levels of dysfunctional eating attitudes, consistent with the proposed intermediary role of body uneasiness in this cross-sectional dataset. This pattern is consistent with the notion that self-reported difficulties in reflective functioning are associated with increased sensitivity to body-related distress and maladaptive eating patterns, although no causal interpretation can be drawn given the cross-sectional design. However, given the cross-sectional design, no directional or causal interpretation can be drawn (Skårderud, 2007; Luyten et al., 2012). These findings extend prior work demonstrating associations between mentalization deficits and eating disorder features (Jewell et al., 2016; Oldershaw et al., 2011; Tonelli and de Siqueira Rotenberg, 2021) by identifying body uneasiness as a potential pathway linking these constructs. However, several important caveats must be acknowledged. The cross-sectional design of the present study precludes any causal or temporal interpretation of these associations. Furthermore, the opposing signs of the total effect (negative) and direct effect (positive) suggest a pattern consistent with inconsistent mediation, in which the mediator may suppress part of the direct relationship. This limits the interpretability of the indirect effect as a straightforward mediational pathway and calls for replication with longitudinal or experimental designs.

From an applied perspective, the present findings highlight the potential relevance of targeting both mentalization capacities and body uneasiness in preventive and therapeutic interventions for adolescents showing higher levels of dysfunctional eating patterns. Should these associations be replicated in longitudinal and intervention research, psychoeducational programs aimed at fostering emotional awareness and reflective functioning might represent one potentially relevant avenue for preventive work with adolescents at risk for dysfunctional eating patterns. In clinical contexts, Mentalization-Based Treatment (MBT; Bateman and Fonagy, 2016) has been proposed as a theoretical framework for addressing eating-related psychopathology; however, whether it would be effective in reducing body uneasiness and dysfunctional eating attitudes in non-clinical adolescent populations remain to be evaluated empirically. These indications are preliminary and should be interpreted with caution given the correlational and cross-sectional nature of the present study. These indications remain preliminary given the correlational nature of the present study and should be further evaluated in intervention research.

### 5.1 Strength and limits

This study analyzed the relationship between self-reported mentalization difficulties, body image distress, and dysfunctional eating attitudes and behaviors, involving a sample of 91 Italian adolescents and adhering to ethical standards and data privacy. However, several limitations must be considered. First, the cross-sectional nature of the study does not allow for establishing causal relationships among the variables examined. Therefore, it is not possible to determine whether difficulties in mentalization precede or follow the development of body image distress and dysfunctional eating behaviors. Second, the use of self-report questionnaires, such as the Body Uneasiness Test (BUT), the Eating Attitudes Test (EAT), and the Mentalization Questionnaire (MZQ), may have introduced response bias due to social desirability, psychological defenses, or limited emotional awareness, factors particularly relevant in adolescents and individuals with mentalization difficulties. An additional limitation concerns the procedure adopted to exclude participants with neurological or psychiatric disorders, which was based on adolescents’ self-report. Self-report screening for psychiatric and neurodevelopmental conditions is considered as a limit in adolescent samples. The absence of formal clinical screening or informant-based corroboration means the non-clinical characterization cannot be verified and should be interpreted with caution. Moreover, although the EAT provides useful screening indications for the higher levels of dysfunctional eating patterns, it is not a specific diagnostic tool, which may limit the ability to draw definitive conclusions regarding the actual presence of such disorders in the sample. A further limitation of the present study concerns the lack of assessment of variables such as ethnicity and body mass index (BMI), which may have influenced the observed results; therefore, future research should incorporate these factors in order to provide a more comprehensive and accurate understanding of the phenomena under investigation. Finally, although the sample was balanced with respect to biological sex (45 females, 46 males), it was relatively small (*n* = 91) and predominantly composed of adolescents attending the fourth year of secondary school, which may limit the generalizability of the findings to more diverse or clinical populations.

For future research, several directions are suggested to overcome these limitations and deepen the understanding of these phenomena. It would be desirable to increase the sample and use more specific and objective assessment tools for eating disorders, such as structured clinical interviews including parents, to confirm the presence of dysfunctional eating behaviors beyond the mere risk identified by the EAT. Additionally, longitudinal studies would be appropriate to explore the temporal evolution of mentalization difficulties and body image distress during adolescence, clarifying whether these elements represent predisposing factors, maintenance factors, or consequences of eating disorders.

Ultimately, the results obtained from this study can serve as a useful foundation for the development of targeted preventive or therapeutic interventions (e.g., psychoeducational programs in schools or mentalization training for adolescents), aiming to strengthen socio-cognitive abilities by addressing not only the modification of symptoms related to eating disorders but also enhancing reflective capacities and reducing emotional difficulties.

## Data Availability

The raw data supporting the conclusions of this article will be made available by the authors, without undue reservation.
